# FAMILY CAREGIVER FACTORS AND HOSPITALIZATION IN DISABLED OLDER ADULTS WITH AND WITHOUT DEMENTIA

**DOI:** 10.1093/geroni/igz038.2708

**Published:** 2019-11-08

**Authors:** Halima Amjad, John Mulcahy, Judith D Kasper, Julia Burgdorf, David L Roth, Kenneth Covinsky, Jennifer L Wolff

**Affiliations:** 1 Johns Hopkins University School of Medicine, Baltimore, Maryland, United States; 2 Johns Hopkins University Bloomberg School of Public Health, Baltimore, Maryland, United States; 3 Johns Hopkins Bloomberg School of Public Health, Baltimore, Maryland, United States; 4 University of California San Francisco, San Francisco, California, United States; 5 Johns Hopkins University, Maryland, United States

## Abstract

Older adults with disabilities commonly rely on family caregivers’ help, yet effects of caregiver factors on patient outcomes are poorly understood. Within this population, dementia is common. Our objective was to evaluate the association between caregiver factors and risk of hospitalization in disabled older adults with and without dementia. We examined 2,589 community-living older adults with mobility/self-care disability and their primary family caregiver in four waves of the National Long-Term Care Survey and National Health and Aging Trends Study. We used Cox proportional hazards models to examine risk of one-year, Medicare claims-derived, all-cause hospitalization as a function of caregiver factors, adjusting for older adult characteristics (sociodemographics, comorbidities, healthcare utilization) and survey year, considering dementia a characteristic of interest. Among disabled older adults, 38% were hospitalized over one year, and 31% had probable dementia. Hospitalization rates were similar for older adults with and without dementia (39.5% and 37.3% respectively); dementia was not associated with hospitalization risk (HR 1.09, 95% CI 0.95-1.26). Older adults demonstrated greater risk of hospitalization if their caregiver was male (HR 1.31, 95% CI 1.10-1.56), new to caregiving (HR 1.61, 95% CI 1.27-2.04 for < 1 year versus ≥ 4 years), or helped with healthcare tasks (HR 1.21, 95% CI 1.04-1.41). The association between most caregiving factors and hospitalization risk did not differ by dementia status. Results suggest that strategies to reduce hospitalization in older adults with disabilities could target select caregivers using similar strategies in populations with and without dementia.

